# Data used for detection and tracking of dynamic objects for visually impaired people

**DOI:** 10.1016/j.dib.2019.104403

**Published:** 2019-08-21

**Authors:** Natal Henrique Cordeiro, Emerson Carlos Pedrino

**Affiliations:** a Federal Institute of São Paulo, Brazil; b Federal University of São Carlos, Brazil

**Keywords:** Dynamic objects, Detection, Segmentation, Tracking, Visually impaired people

## Abstract

This paper presents in detail the methodology for the detection and tracking of dynamic objects from the article in press (A new methodology applied to dynamic object detection and tracking systems for visually impaired people [1]). In order to validate this methodology, four different architectures have been designed in this paper. These architectures have implemented the techniques of pattern recognition, optical flow, background subtraction and color tracking to enable comparison and to see which is the most appropriate in a given environment. In this paper we also present a method created to quantify the effectiveness of each architecture implemented.

**Table:** Specifications Table

Subject area	Computer science
More specific subject area	Computer Vision and Image Processing
Type of data	Image, graph, video and spreadsheet
How data was acquired	Microsoft Kinect RGB-D (Infrared and RGB camera) matrices
Data format	eps, pdf, avi, xls
Experimental factors	Data from the infrared and RGB sensor were converted into appropriate matrices with RGB channels.
Experimental features	Images produced for Detection and Tracking of Dynamic Objects and figures that detail the applied methodology in order to calculate effectiveness.
Data source location	Indoor environment (SP-Brazil)
Data accessibility	Image, video and spreadsheet (Supplementary files)
Related research article	Natal Henrique Cordeiro, Emerson Carlos Pedrino, A new methodology applied to dynamic object detection and tracking systems for visually impaired people, Computers & Electrical Engineering, Volume 77, 2019, Pages 61–71, ISSN 0045–7906, https://doi.org/10.1016/j.compeleceng.2019.05.003.

**Table:** 

**Value of the data** • The data enable visually identifying which detections were effectively mapped using dynamic object detection and tracking techniques. • The data allows (researcher) the chance of measuring the and effectiveness of each technique used for detection and tracking of dynamic objects. • The data allow to understand how the detection of a dynamic object and error identification is performed by each technique. • These data demonstrate how the positions of the detected dynamic objects can be mapped in millimeters. • The quality of segmentation performed by each technique can be understood visually.

## 1 Data

**Fig. 1:**
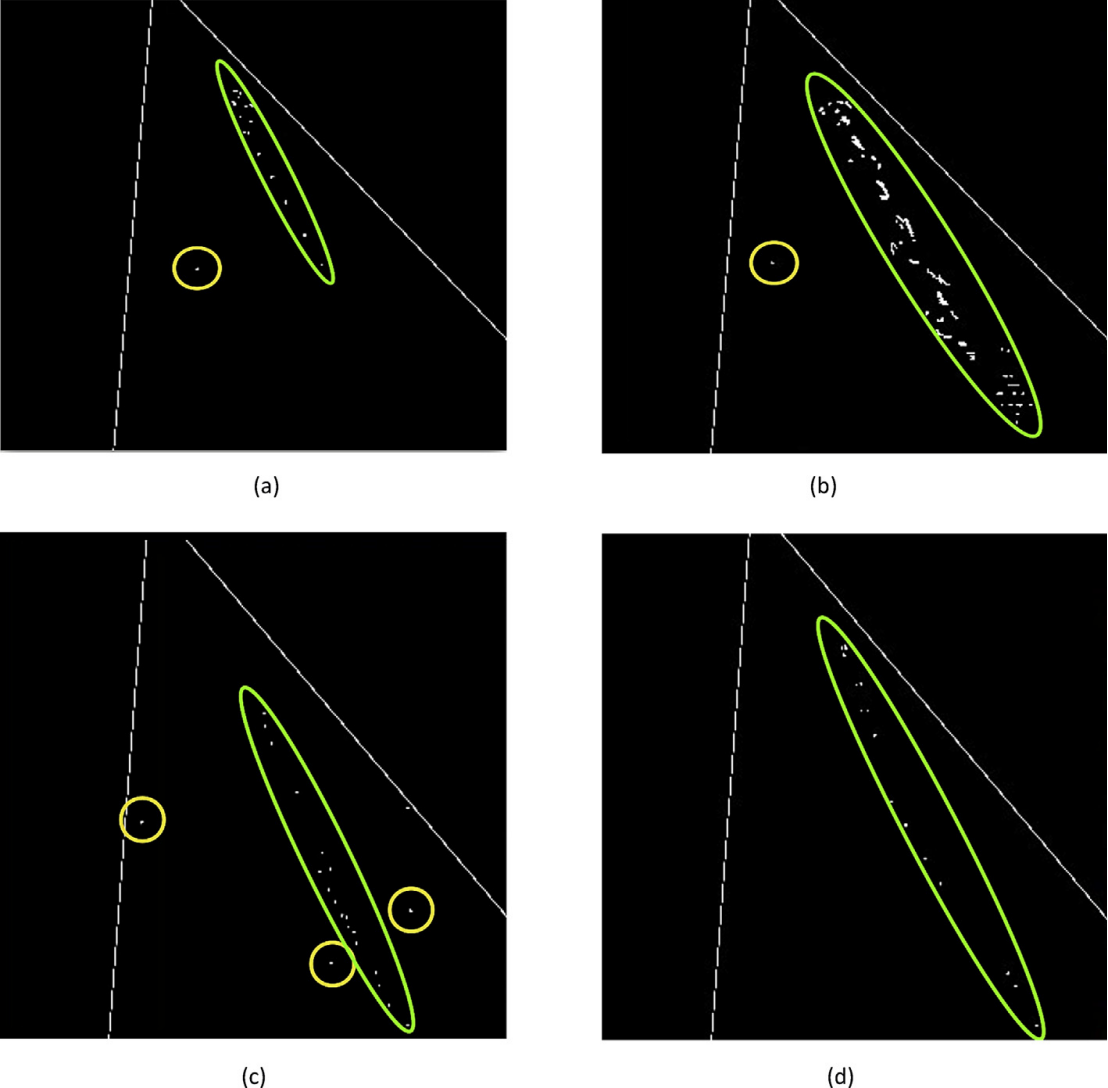
Measuring the effectiveness using MAP (X,Z).

**Fig. 2:**
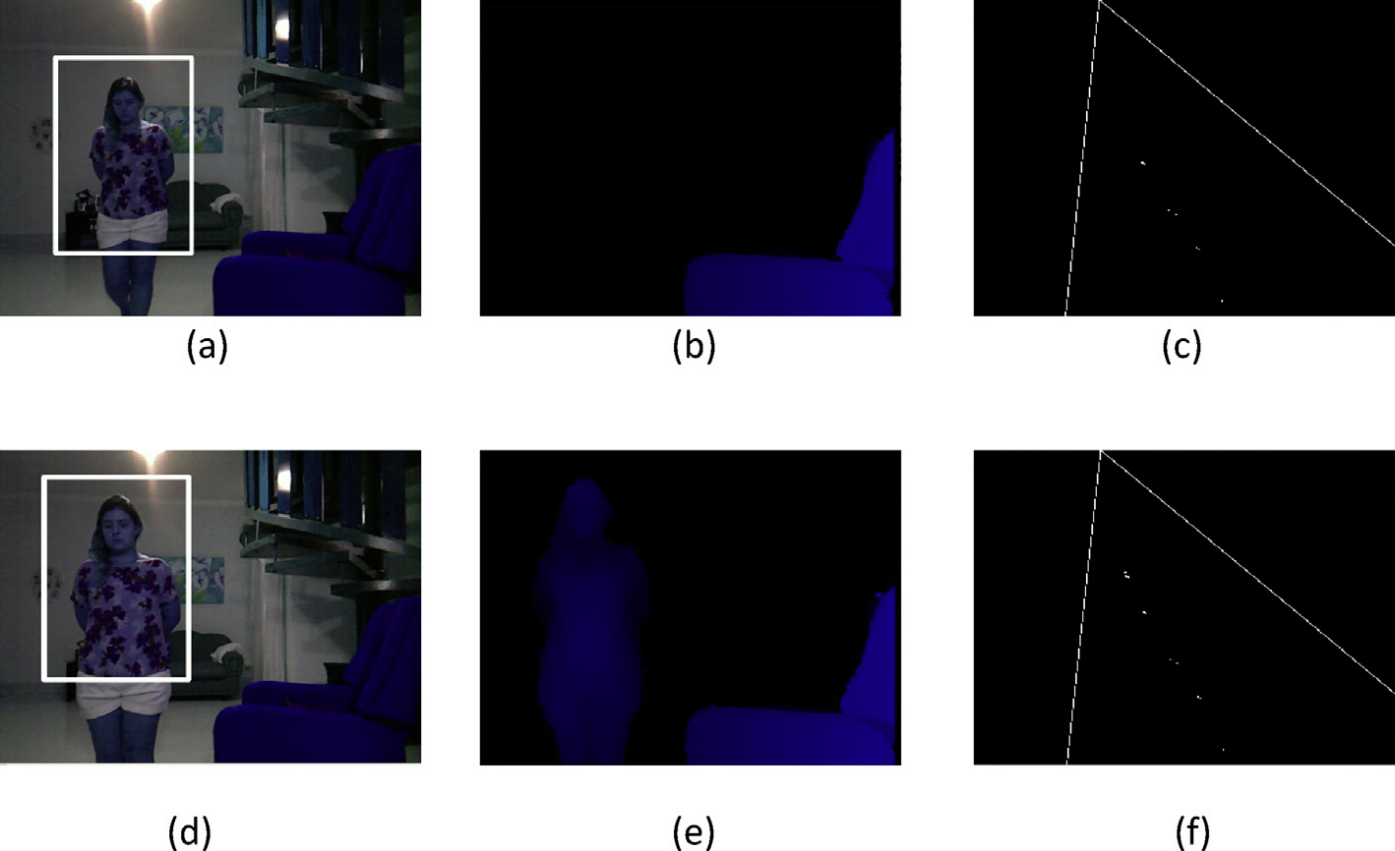
Detection and Tracking of DO using the Architecture A1-PR.

**Fig. 3:**
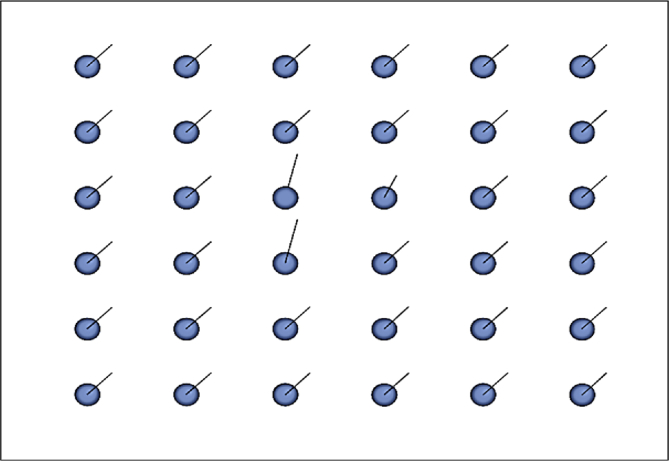
Dense map (Farneback).

**Fig. 4:**
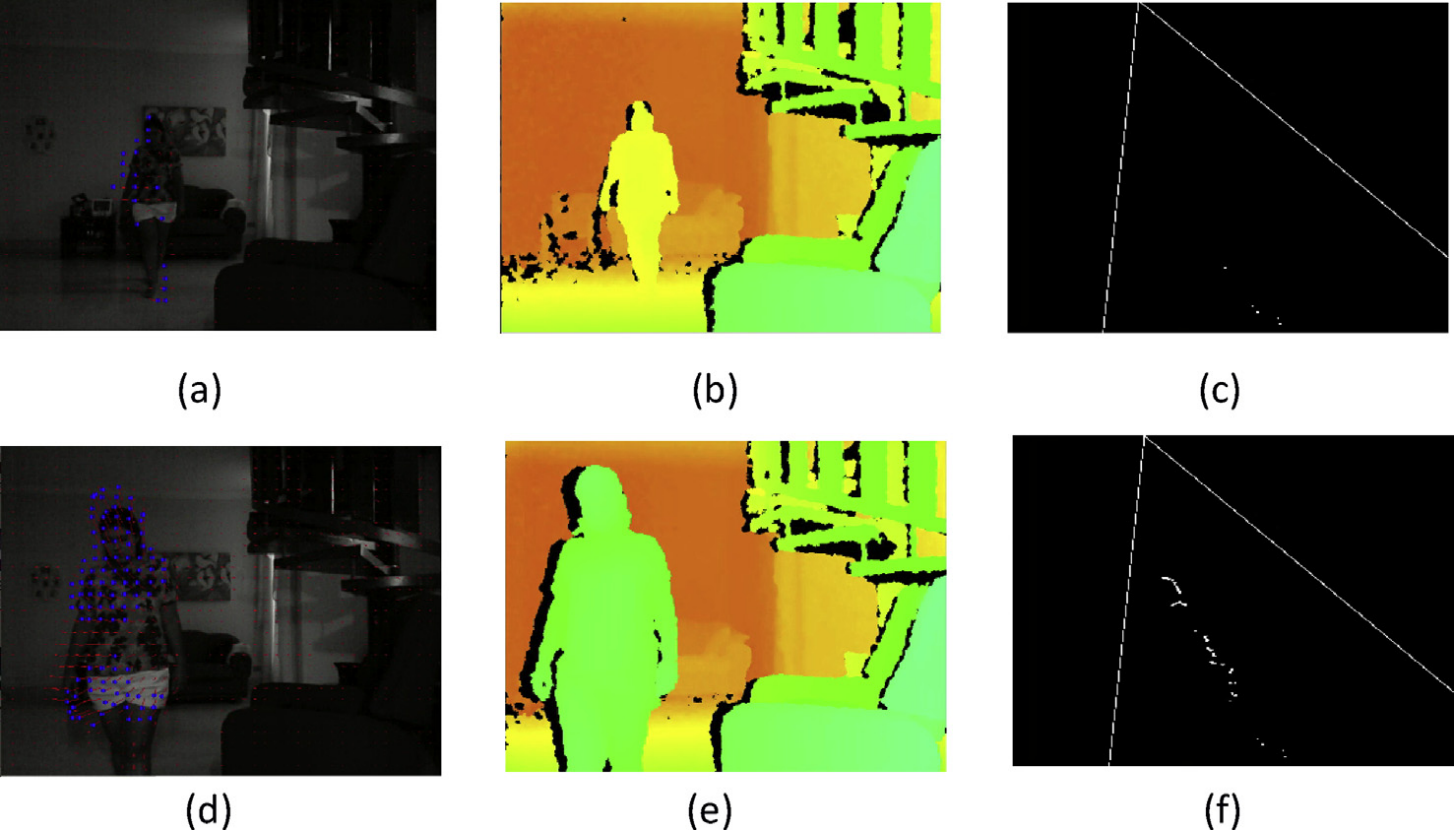
Detection and Tracking of dynamic object using the Architecture A2-FOF.

**Fig. 5:**
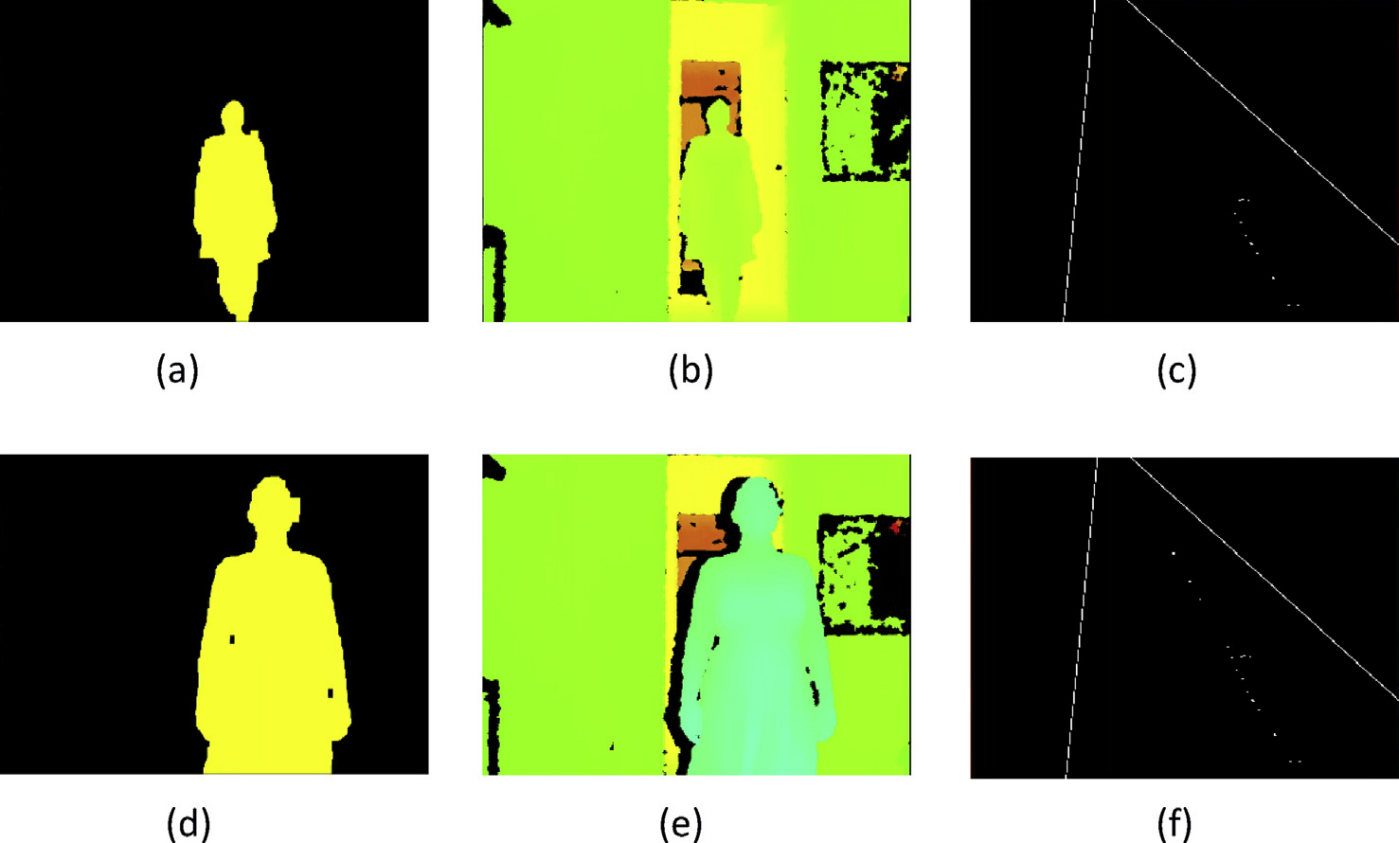
Detection and Tracking of DO using the Architecture A3-BS.

**Fig. 6:**
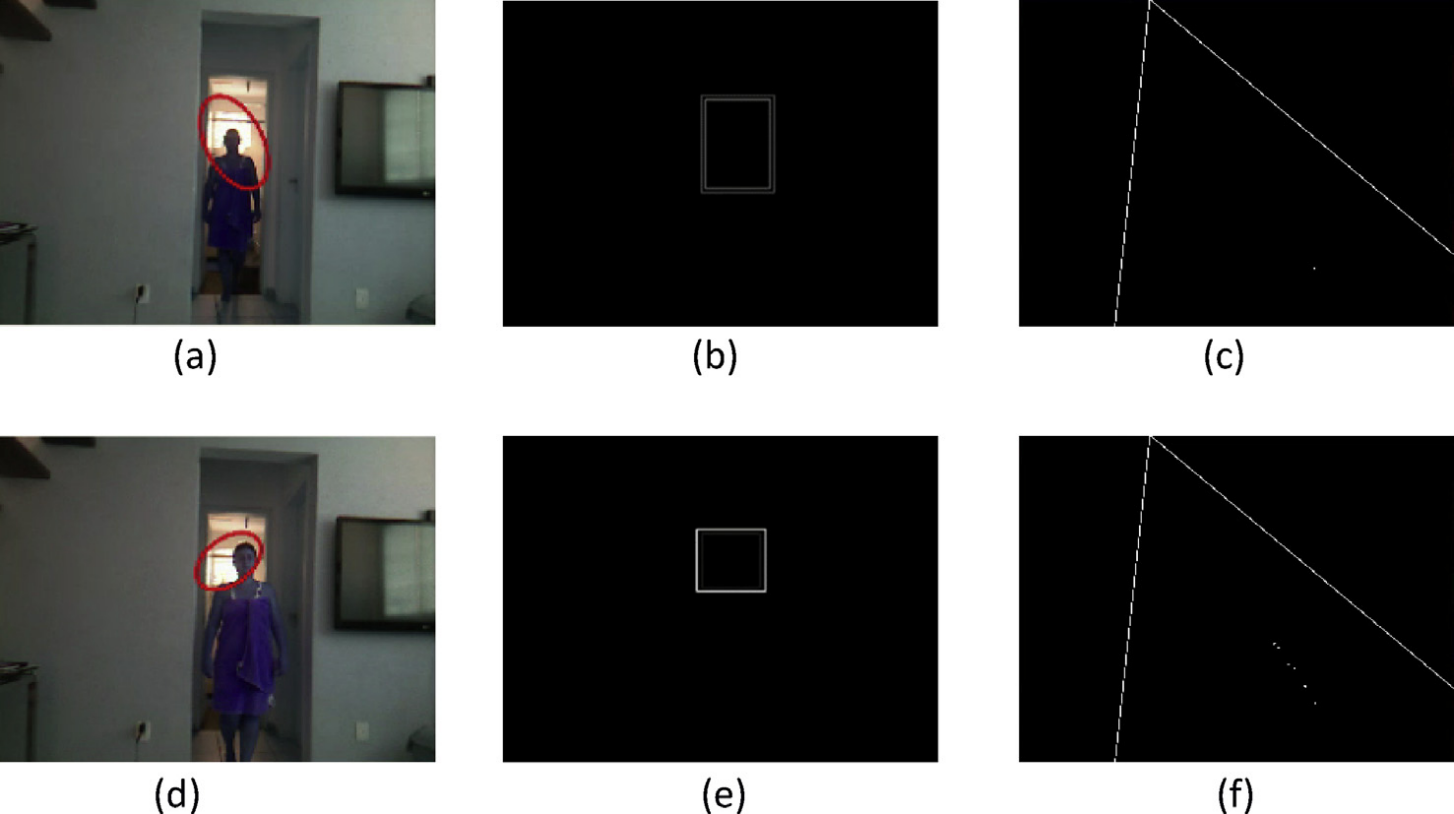
Detection and Tracking of dynamic object using the Architecture A4-CamShift.

**Fig. 7:**
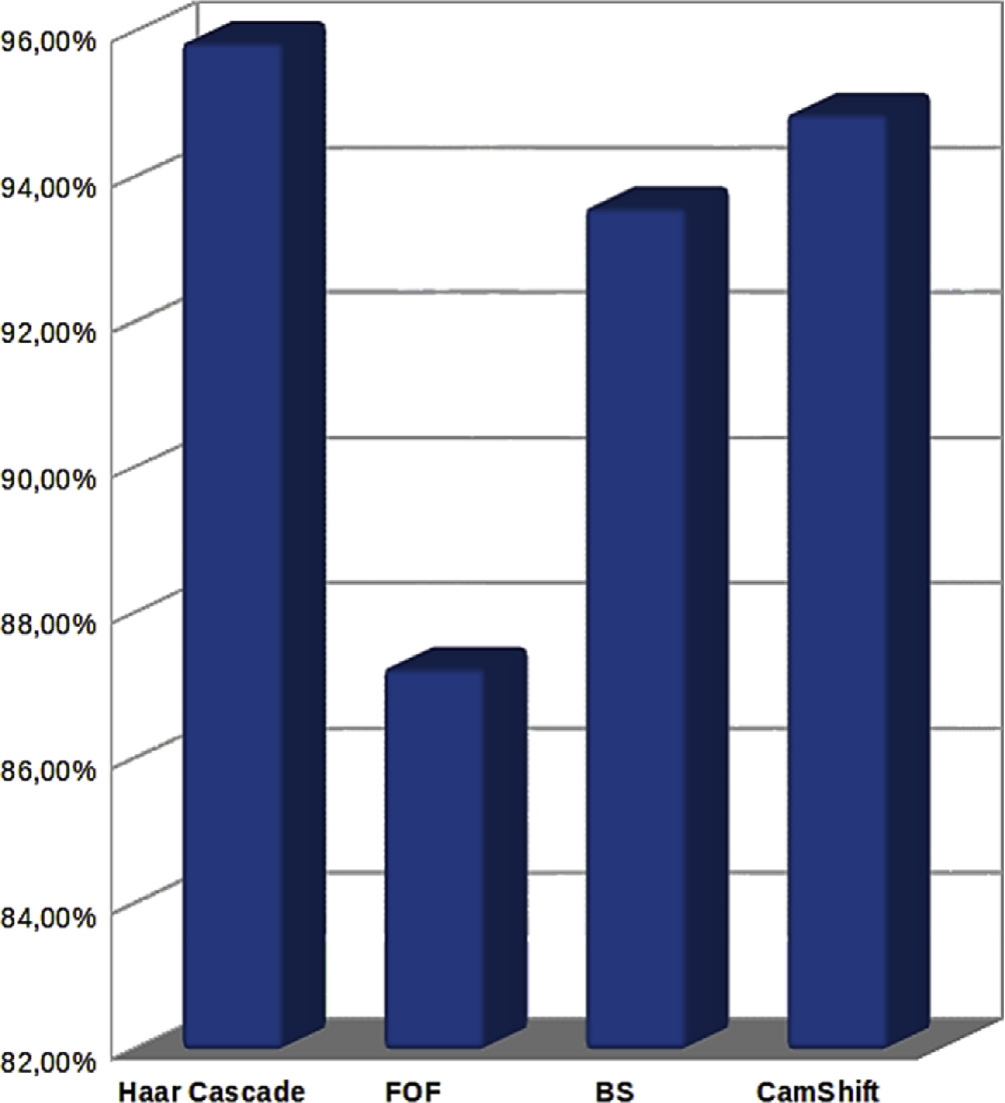
Dynamic object detected and mapped correctly.

These data present a methodology applied to compare four techniques for the detection and tracking of dynamic objects (DTDO) regarding their effectiveness. The implemented techniques were customized from operational architectures. Fig. 1 describes the methodology applied to the analysis of the effectiveness of each technique. The techniques used in this context were: 1- Pattern Recognition (PR); 2- Farneback Optical Flow (FOF); 3- Background Subtraction (BS); and 4- Continuously Adaptive Meanshift (CamShift). Fig. 2 depicts two examples of the DTDO method using the PR technique. Fig. 3 shows part of the detection from the processing of the dynamic objects implemented by the FOF technique. Algorithm 1 outlines the adaptation that has been made to detect dynamic objects by using FOF even when movements of the acquisition sensor take place. Fig. 4 depicts two examples of the DTDO method by using the FOF technique. Fig. 5 illustrates two examples of DTDO by using the BS technique. Fig. 6 displays two examples of DTDO by using the CamShift technique. Fig. 7 depicts a graph containing the effectiveness of each technique. Details of the implementation of these four architectures can be seen in the main article previously published. (A new methodology applied to dynamic object detection and tracking systems for visually impaired people [1]).

### 1.1 Data acquisition for detection and tracking of dynamic objects

The data presented in this article show that the architectures acquire the necessary data for the 3D tracking and mapping of the dynamic objects. The methodology applied in the detection process to account for effectiveness is also given. All modules present in the architectures were implemented with the help of the OpenCV library [2]. In this project, the Microsoft Kinect RGB-D was placed at the front of the waist of the VIP. When a dynamic object is detected, regardless of speed, a beep will be emitted. However, tracking may not be to the expected quality due to the frame capture rate and processing speed is low relative to that of the moving object.

Hence the importance of analyzing the speed of detection (SpD) of each architecture projected. SpD allows observing which architecture produces the longest run time to trigger a beep (see supplementary file - spreadsheet). Each beep indicates the position of the dynamic object detected. This comparison is independent on the processing power used. Effectiveness is another and more important feature the data (Maps) of this study allows calculating. The analysis of which detections were performed improperly is conducted based on effectiveness. This occurs when noises are generated by changes in lighting, strong movement in the acquisition sensor, or even when objects similar to those programmed for the techniques of pattern (PR) or color recognition (CamShift) are detected in the environment. Two indoor environments with different types of lighting, objects and passages were defined. For each environment, detection and tracking of the dynamic object were performed using the four architectures. For each architecture, six paths were reconstructed in each environment. Of these reconstructions, three had a static camera and the other three had camera movements. Thus, forty eight paths were obtained to perform a comparative analysis between the architectures implemented. Based on the reconstruction of the paths, the quality of detection and tracking of the dynamic object could be analyzed the efficiency of four approaches.

## 2 Experimental design, materials and methods

In Fig. 1, the data (images) show the methodology applied to compare the four architectures with respect to the effectiveness. In these images (Map (X, Z)) the amount of detection (blank points) performed until the end of the tracking is shown. The green circles show the tracking of the dynamic object until its completion. As the system records the start and end times, the number of detections carried out per second can be calculated at the end of each tracking, for each architecture. This map also allows for error identification during the detection process (Effectiveness). In the images in Fig. 1, the yellow circles represent errors in the detection process. The images (a, b, c, d) show the following scans: Architecture 1 - Pattern Recognition (A1-PR), Architecture 2 - Farneback Optical Flow (A2-FOF), Architecture 3 - Background Subtraction (A3-BS) and Architecture 4 - Continuously Adaptive Meanshift (A4-CamShift) respectively. The spreadsheet containing the raw data obtained to compare the effectiveness of each architecture implemented can be seen in Supplementary files (spreadsheet 1). Each point rendered on the map (X, Z) means that a beep was sounded to the VIP to show the position of the dynamic object in the environment.

In the images in Fig. 1, observing that experiments were carried out in the same region is possible. Even so, the points (blank points) allow observing that each architecture shows differences in the process of detection and tracking of the dynamic object.

The details of the experiments are divided into four topics. Each topic provides the input images, the segmented objects and the map (X, Z) used to compare effectiveness.

### 2.1 Architecture 1 - pattern recognition (A1-PR)

Fig. 2 (images (a – f)) show two instances of data acquisition for detection and tracking of dynamic object using Architecture 1 - Pattern Recognition. In this architecture, the detection was performed using the Haar Feature technique based on Cascade classifiers [3]. These acquisitions occur with the camera suffering little movement (see supplementary video and tab A1-PR in spreadsheet 1). Each sequence is composed of the following images: dynamic object detection; the depth map that only shows objects at less than 2 m; the position of the dynamic object on the map (X, Z) at the same instant as its detection.

The following are the supplementary data related to this article:

Video 1 Video 1

Video 2 Video 2

### 2.2 Architecture 2 - Farneback Optical Flow (A2-FOF)

The Farneback Optical Flow method was the only one adapted regarding the way of detecting dynamic objects. These adaptations were made to detect the dynamic object even faced with movements in the acquisition sensor (IR and RGB sensors). The first step of this technique is to distribute a set of points that will determine the optical flow between subsequent frames. These can be seen in Fig. 3 and are created in Algorithm 1 (lines 3 and 4).

For the FOF technique to detect the dynamic object, vectors were created for each point in the image (line 7). These vectors represent the velocity and direction respectively by their size and their angle (see supplementary files – image 5). The larger the vector, the greater the flow detected at a certain point on the map. The size and angles of the vectors allow us to differentiate which are movements of the acquisition sensor and which are movements of a dynamic object. Because all vectors reproduce changes when the camera is moved, the vector sizes were averaged using Euclidean distances (line 8). In the same way, the mean (line 9) of the angles produced with these movements was created. The angles were generated by the function **atan2** and the parameters are the same as those already given. Thus, for each point, a check was made to see if there was a change of flow and direction above the average.

When the acquisition sensor undergoes some movement or when a dynamic object is detected, the vectors have their size modified. By analyzing the mean it is possible to verify the points that have suffered major flows. Likewise, when there is camera movement, the angles are similar. However, if any angle has a high divergence with respect to the mean, the probability of a dynamic object being detected is large (lines 13 and 14). For this method, besides the averages, the concepts of covariance (line 15) were also implemented for the size (**Cov**_**Euclidian**_ > **value**_**1**_) and the direction (**Cov**_**Angle**_ > **value**_**2**_) of the vectors produced, where values (**value**_**1**_ and **value**_**2**_) are the thresholds found (average) and differ when the flow detected at one point is more than the others.

Algorithm 1 Adaptation to detect dynamic object using FOF even with movements of the acquisition sensor).

**Figure:**
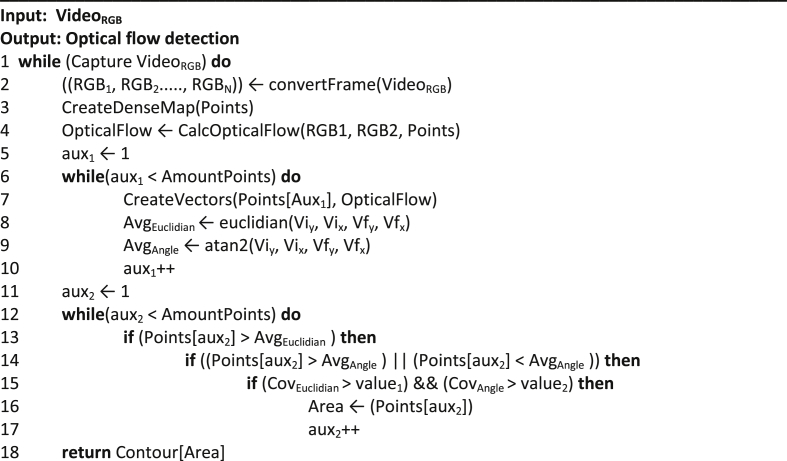

Fig. 4 (images (a – f)) show two instances of data acquisition for Detection and Tracking of dynamic object using Architecture 2 - Farneback Optical Flow. These acquisitions occur with the camera suffering little movement (see supplementary video and tab A2-FOF in spreadsheet 1). Each sequence is composed of the following images: dynamic object detection; the depth map that provides the distances; the position of the dynamic object on the map (X, Z) at the same instant as its detection.

### 2.3 Architecture 3 - background subtraction (A3-BS)

Fig. 5 (images (a – f)) show two instances of data acquisition for detection and tracking of dynamic object using Architecture 3 - Background Subtraction. These acquisitions occur with the camera suffering little movement (see supplementary video and tab A3-BS in spreadsheet 1). Each sequence is composed of the following images: dynamic object detection using BS; the depth map that provides the distances; the position of the dynamic object on the map (X, Z) at the same instant as its detection.

### 2.4 Architecture 4 - Continuously Adaptive Meanshift (A4-CamShift)

Fig. 6 (images (a – f)) show two instances of data acquisition for detection and tracking of dynamic objects using Architecture 4 - Continuously Adaptive Meanshift. This architecture performs single hue object tracking. The acquisitions occur with the camera suffering little movement (see supplementary video and tab A4-CamShift in spreadsheet 1).

Each sequence is composed of the following images: dynamic object detection; the outline of the image (a); the position of the dynamic object on the map (X, Z) at the same instant as its detection.

With the set of paths reconstructed, a comparative analysis could be done between some characteristics of the four detection and tracking architectures being tested. From all the data related to the effectiveness for each architecture was generated. The videos of the experiments conducted, the images of the maps (X,Z), and the data for analysis of effectiveness are available in Supplementary files (tab effectiveness in spreadsheet 1). Fig. 7 present the averages obtained (effectiveness) for each architecture.
