# Supplementary figures and images for: Data used for detection and tracking of dynamic objects for visually impaired people
Source: Data Brief. 2019 Aug 21;26:104403. doi: 10.1016/j.dib.2019.104403 (PMC6728795; doi:10.1016/j.dib.2019.104403)

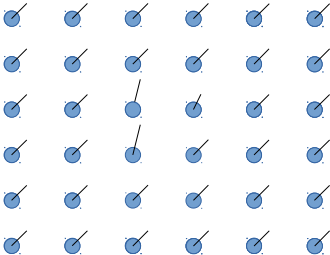

Supplement: Video 1 [file mmc2.zip › Supplementary_images/3.pdf]
